# Supplementary material for: Predicting mechanical ventilation effects on six human tissue transcriptomes
Source: PLoS One. 2022 Mar 10;17(3):e0264919. doi: 10.1371/journal.pone.0264919 (PMC8912236; doi:10.1371/journal.pone.0264919)
Supplement: S1 File — (PDF) [file pone.0264919.s001.pdf]

Supplemental File

Predicting mechanical ventilation and its effects on human tissue transcription

Judith Somekh[a], Nir Lotan[a], Ehud Sussman[a], Gur Arye Yehuda[a]

[a] Department of Information Systems, University of Haifa, Haifa, Israel

Correspondence email: judith_somekh@is.haifa.ac.il

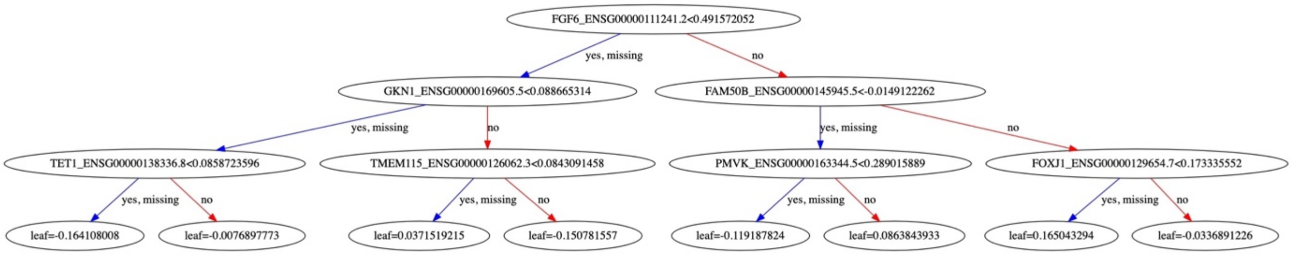

Figure S1. Sample XGBoost tree branch in muscle-skeletal tissue.

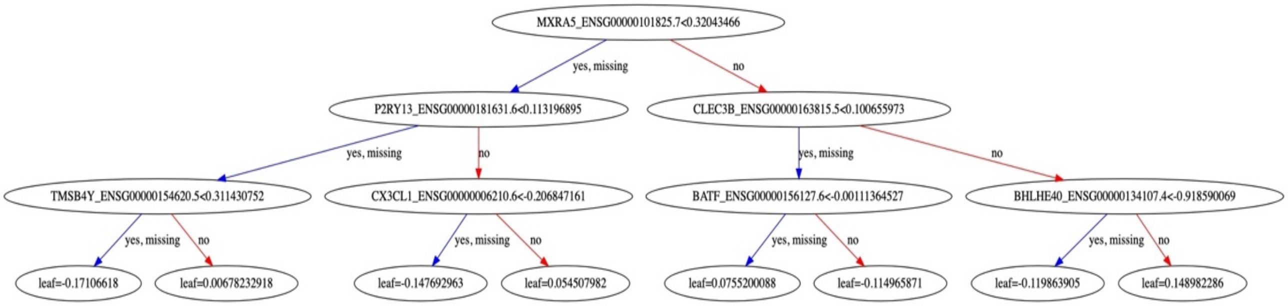

Figure S2: Sample XGBoost tree branch in adipose-subcutaneous.

Table S1. Number of samples and features per tissue.

Tissue	# of samples	# of ventilation	# of non-ventilation	# of features
Adipose-subcutaneous	544	349	195	16,052
Liver	204	119	85	15,268
Lung	476	297	179	16,534
Muscle-skeletal	650	417	233	14,845

Tissue	# of samples	# of ventilation	# of non-ventilation	# of features
Nerve-tibial	506	322	184	12,978
Skin lower leg (sun exposed)	565	348	217	16,442

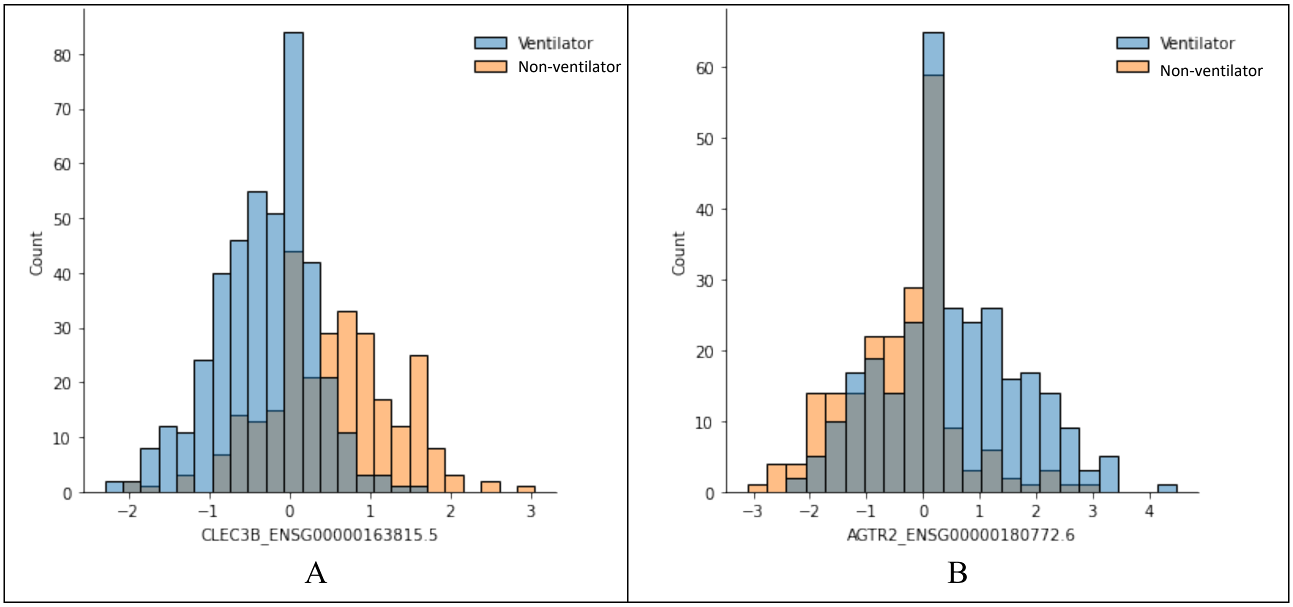

Figure S3. Histograms illustrating specific gene values in different tissues for the ventilation and non-ventilation types. After detecting the relevant genes per tissue type, we plot a histogram for the most discriminant genes per the relevant tissue. (A) Histogram illustrating the values of CLEC3B in muscle-skeletal tissue, in which low values of the gene appear mostly among individuals receiving ventilation. (B) The values of AGTR2 in lung tissue, in which low values of the gene appear mostly among the non-ventilation group.

Table 2. Performance results of 18 models, applying three machine learning methods—XGBoost, RF, NN—on six tissues.

Model	Organ	AUC	AUC K-fold std	Accuracy	Accuracy K-fold std	F1_score	F1 Score K-fold std	Recall	Recall K-fold std	Precision	Precision K-fold std
**Random Forest**	**Adipose-subcutaneous**	0.863	0.038	0.879	0.032	0.827	0.047	0.807	0.074	0.853	0.058
**Random Forest**	**Liver**	0.952	0.040	0.953	0.039	0.957	0.037	0.962	0.068	0.958	0.060
**Random Forest**	**Lung**	0.932	0.034	0.933	0.031	0.912	0.040	0.928	0.053	0.899	0.044
**Random Forest**	**Muscle-skeletal**	0.917	0.043	0.931	0.032	0.898	0.052	0.870	0.087	0.932	0.042
**Random Forest**	**Nerve-tibial**	0.891	0.033	0.913	0.025	0.869	0.041	0.809	0.069	0.945	0.045
**Random Forest**	**Skin-sun exposed (lower leg)**	0.852	0.040	0.864	0.036	0.818	0.049	0.799	0.067	0.843	0.053

Model	Organ	AUC	AUC K-fold std	Accuracy	Accuracy K-fold std	F1_score	F1 Score K-fold std	Recall	Recall K-fold std	Precision	Precision K-fold std
**XGBoost**	**adipose-subcutaneous**	0.944	0.036	0.950	0.032	0.930	0.046	0.919	0.054	0.943	0.046
**XGBoost**	**Liver**	0.957	0.042	0.958	0.042	0.961	0.040	0.962	0.068	0.966	0.059
**XGBoost**	**Lung**	0.951	0.031	0.954	0.027	0.939	0.037	0.939	0.049	0.940	0.039
**XGBoost**	**Muscle-skeletal**	0.939	0.040	0.949	0.030	0.926	0.048	0.905	0.084	0.953	0.046
**XGBoost**	**Nerve-tibial**	0.947	0.039	0.957	0.030	0.937	0.046	0.913	0.074	0.966	0.030
**XGBoost**	**Skin-sun exposed (lower leg)**	0.934	0.036	0.940	0.034	0.921	0.045	0.908	0.057	0.937	0.054
**Neural Net**	**Adipose-subcutaneous**	0.935	0.024	0.939	0.018	0.916	0.027	0.918	0.043	0.914	0.010
**Neural Net**	**Liver**	0.926	0.040	0.931	0.038	0.940	0.032	0.971	0.014	0.911	0.048
**Neural Net**	**Lung**	0.939	0.033	0.941	0.024	0.922	0.034	0.928	0.071	0.918	0.001
**Neural Net**	**Muscle-skeletal**	0.937	0.036	0.940	0.024	0.916	0.037	0.927	0.079	0.907	0.004
**Neural Net**	**Nerve-tibial**	0.937	0.021	0.937	0.011	0.914	0.019	0.940	0.055	0.892	0.013
**Neural Net**	**Skin-sun exposed (lower leg)**	0.911	0.042	0.913	0.033	0.888	0.046	0.904	0.084	0.875	0.010

Detailed confounding factor adjustment

Our aim was to correct for multiple confounding factors, among them time (ischemic time), without harming the ventilation signal that we used for our prediction models. When we corrected the data for multiple confounding factors including ischemic time, we detected a discrepancy. It stemmed from the correlation between ischemic time and the ventilation signal. It is common knowledge and was shown that RNA levels degrade over time and their gene expression levels decrease over time. When we corrected the data for known confounding factors using a linear regression model including ischemic time as a predictor, we detected genes which levels increased with time. We investigated this phenomenon to conclude that the uncorrected ventilation signal affects the corrected time predictor coefficient since time and ventilation type are correlated.

Figure S3A shows an example of an erroneous positive time coefficient for the ENSG00000186815.5 gene in muscle-skeletal tissue. If ventilation type (death type 0) and non-ventilation (death type 1) are assumed to be the same, one would naively get a positive regression line (shown in yellow) for the gene expression level of that particular gene as a function of ischemic time. Since gene expression levels degrade over time, these results are skewed and affected by the correlated biological signal related to ventilation/non-ventilation. The result of the

yellow lined slope represents a growth in the gene expression rate associated with the time of 0.001359, with a t value of 15.960 and p value of 0.000. Accordingly, the time predictor was eliminated and no further adjustments for ischemic time were performed for genes whose expression levels increased over time (since we assume that this increase is not related to time).

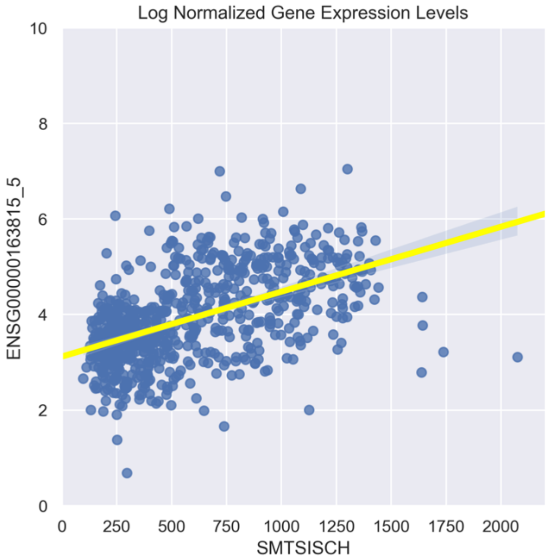

Figure S4. The ischemic growth rate, which is well beyond statistical tolerances, if samples are not split by death type.

We took a different approach for genes where the time coefficient was negative as expected. For example, within the muscle-skeletal group, gene ENSG00000165424.6 demonstrated the second highest predictive significance, for ventilation type. In this instance we do see the expected negative slope of gene expression rates as a function of ischemic times; however, after a more detailed analysis, it appears that the ischemic prediction is overstated (see Figure S4). When death types are not separated, we get an ischemic rate coefficient of -0.000938, a $t$value of -13.413 with a corresponding p value of 0.000.

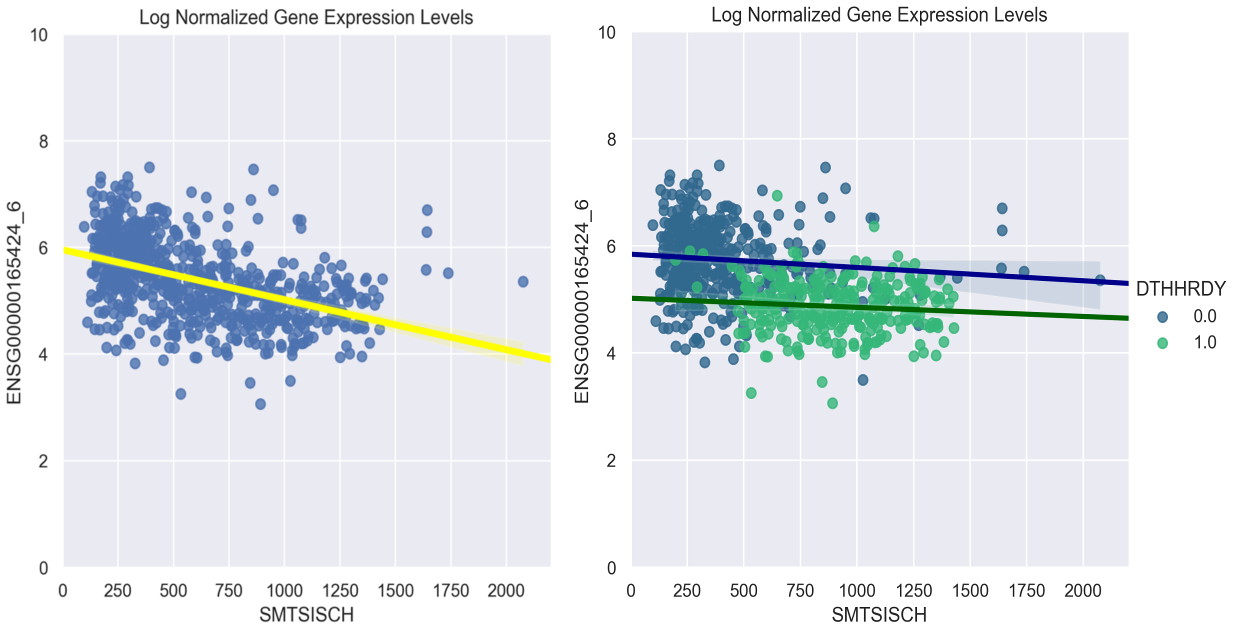

Figure S5. (A) An example of an ischemic rate of decay that is overestimated, if the samples are not split by death type. (B) The samples are bifurcated by death type and we still observe ischemic decay, albeit a more moderate rate of decay.

When death types are separated, for the ventilator cases, indicated by the dark blue line, we get an ischemic rate of -0.000248 with a t value of -1.959 and a p value of 0.051. For the non-ventilator cases, indicated by the dark green line, we have an ischemic rate of -0.000170, a t value of -1.491 and a p value of 0.137. We then average the ischemic rates to get a combined rate of -0.000209. If we applied the alternative 2 methodology, the combined ischemic rate would have yielded -0.000217.

We performed this linear regression in two steps, as described in the main part of the article.

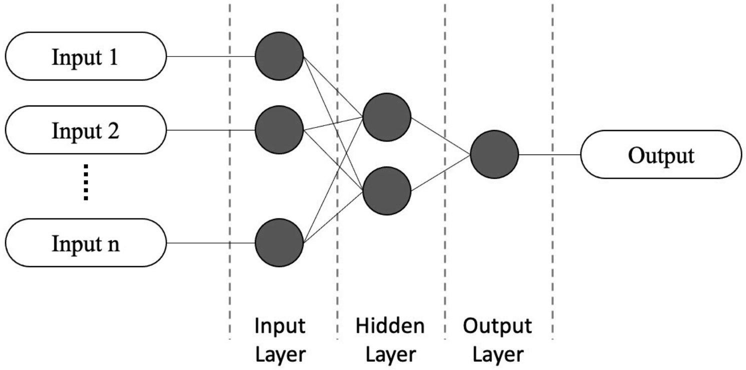

Figure S6. The selected neural network topology

Table S3. Enrichment analysis of features in subcutaneous adipose.

Term	P-value	Adjusted P-value	Genes

Viral protein interaction with cytokine and cytokine receptor	5.81E-08	1.59E-05	IL10;CXCL9;CCL22;CXCR4;CXCL1;CXCL13;CXCL3;TNF;CXCL2;CX3CL1;IL22RA1;CCL8;CXCR1;CXCR3;XCL2;ACKR4;ACKR3;CCR10;CCL27;CCR2
Cytokine-cytokine receptor interaction	1.02E-06	1.40E-04	CXCL9;CTF1;IL25;EBI3;CXCR4;CXCL1;CXCR6;CXCL13;CXCL3;TNF;CXCL2;CX3CL1;CCL8;CXCR1;CXCR3;CCR10;CCR2;IL10;CCL22;TNFSF15;LIF;BMP8A;NGF;BMP6;IL22RA1;BMP3;BMP2;IFNG;XCL2;ACKR4;ACKR3;IL17D;INHA;CCL27;TNFRSF21
Amoebiasis	1.17E-04	0.0107231	IL10;HSPB1;CXCL1;SERPINB9;CD1D;LAMC1;CXCL3;TNF;CXCL2;CD1A;GNA14;GNA15;IFNG;CTSG;TLR2
Chemokine signaling pathway	4.39E-04	0.03004752	CXCL9;CCL22;CXCR4;CXCL1;CXCR6;CXCL13;GNG12;CXCL3;GNG11;CXCL2;CX3CL1;GNG10;CCL8;CXCR1;CXCR3;XCL2;GNG8;CRK;CCR10;CCL27;CCR2
Asthma	0.00334344	0.17403464	IL10;HLA-DRB5;RNASE3;EPX;TNF;HLA-DQA2
Glycosaminoglycan biosynthesis	0.00381098	0.17403464	CHST7;HS3ST3A1;CHSY1;B3GNT2;CHSY3;HS6ST1;CHST3;HS3ST2
Neuroactive ligand-receptor interaction	0.00482278	0.18877754	PTGER4;GLP1R;FPR3;OXT;GRPR;ADRA1B;GALR2;HRH4;CTSG;DRD1;INSL3;PTGDR;NTSR2;P2RY13;P2RY11;GZMA;F2R;SLURP1;GABRG1;GRIN2D;GRIN3B;GRIN3A;SST;NMUR2;RLN3;NMUR1;F2RL2;F2RL3
Inflammatory bowel disease	0.01305771	0.44722671	IL10;HLA-DRB5;MAF;IFNG;TBX21;TNF;HLA-DQA2;TLR2
Rheumatoid arthritis	0.01469527	0.44738937	HLA-DRB5;IFNG;MMP1;CXCL1;CXCL3;CXCL2;TNF;ATP6V0D2;HLA-DQA2;TLR2
Staphylococcus aureus infection	0.0168674	0.46216689	IL10;HLA-DRB5;DEFA6;DEFA4;KRT25;KRT14;DEFB1;FPR3;KRT31;HLA-DQA2
Intestinal immune network for IgA production	0.02782777	0.69316455	IL10;PIGR;HLA-DRB5;CXCR4;HLA-DQA2;CCR10
Allograft rejection	0.03552878	0.72728975	IL10;HLA-DRB5;IFNG;TNF;HLA-DQA2
IL-17 signaling pathway	0.0388016	0.72728975	IL25;IFNG;MMP1;CXCL1;CXCL3;IL17D;CXCL2;TNF;MMP9
TGF-beta signaling pathway	0.0388016	0.72728975	GREM2;LEFTY1;BMP2;IFNG;NOG;BMP8A;ID3;TNF;BMP6
Fatty acid elongation	0.03981513	0.72728975	ECHS1;ELOVL2;ELOVL3;ACOT4
Legionellosis	0.05731508	0.98152073	PYCARD;CXCL1;CXCL3;CXCL2;TNF;TLR2

Table S4. Enrichment analysis of features in liver.

Term	P-value	Adjusted P-value	Genes
Cytokine-cytokine receptor interaction	7.41E-11	1.96E-08	BMP10;TNFRSF6B;MSTN;IL27;CXCL1;CXCR6;CXCL13;CXCL3;CXCL2;CX3CL1;CXCR3;CCR7;CCR4;CCR10;PF4V1;CCR2;IL10;CCL22;TNFSF15;LIF;OSM;INHBB;PPBP;NGF;BM

			P6;INHBE;TNFRSF10D;BMP2;IFNG;LEP;XCL2;ACKR4;CD27;XCL1;PF4
Viral protein interaction with cytokine and cytokine receptor	6.78E-10	8.94E-08	IL10;CCL22;CXCL1;PPBP;CXCL13;CXCL3;CXCL2;CX3CL1;TNFRSF10D;CXCR3;XCL2;ACKR4;XCL1;CCR7;CCR4;CCR10;PF4V1;CCR2;PF4
Chemokine signaling pathway	2.34E-05	0.00205689	CCL22;CXCL1;PPBP;CXCR6;CXCL13;GNG12;CXCL3;CXCL2;CX3CL1;PIK3CA;CXCR3;XCL2;XCL1;CCR7;CCR4;CCR10;PF4V1;CCR2;PF4
Neuroactive ligand-receptor interaction	2.02E-04	0.01332367	PTGER1;HRH1;GALR3;NPBWR1;CHRNE;HRH4;GNRHR;S1PR1;CTSG;S1PR3;DRD1;INSL3;NTSR1;DRD4;PTGDR;P2RY13;SSTR1;GRIN2D;GRIN3B;GRIN3A;ADORA2B;SST;LEP;AVP;F2RL2
Amoebiasis	0.002105653	0.1111785	IL10;GNA15;IFNG;PIK3CA;HSPB1;CXCL1;CTSG;CXCL3;CXCL2;TLR2
Rheumatoid arthritis	0.012253532	0.5391554	ATP6V1G1;IFNG;CXCL1;CXCL3;CXCL2;HLA-DQA2;TLR2;ATP6V1F
Asthma	0.018692896	0.70498923	IL10;RNASE3;EPX;HLA-DQA2
cAMP signaling pathway	0.028972006	0.95607619	NPR1;SSTR1;GRIN2D;HCAR2;HCAR3;GRIN3B;GRIN3A;RRAS;PIK3CA;SST;PDE3A;FFAR2;DRD1
Steroid hormone biosynthesis	0.051868873	0.9997781	HSD11B2;CYP1A2;UGT2B17;CYP7B1;HSD17B8

Table S5. Enrichment analysis of features in lung.

Term	P-value	Adjusted P-value	Genes
Cytokine-cytokine receptor interaction	1.49E-09	4.12E-07	CXCL9;TNFRSF6B;CCL11;IL25;IL26;EPO;EBI3;CXCR5;IL27;CXCL1;CXCL13;TNF;CXCL2;CX3CL1;CXCL5;IFNL1;CXCR3;CCR4;CCR10;CCR2;IFNA21;IL10;TNFSF15;GDF15;LIF;BMP8A;OSM;GDF3;PPBP;INHBC;BMP6;IL2;INHBE;IFNG;LEP;IFNK;XCL2;ACKR4;ACKR3;LTB;IL17D;INHA;IL17B
Viral protein interaction with cytokine and cytokine receptor	1.05E-06	1.45E-04	IL10;CXCL9;CCL11;CXCR5;CXCL1;PPBP;CXCL13;TNF;CXCL2;IL2;CX3CL1;CXCL5;CXCR3;XCL2;ACKR4;ACKR3;CCR4;CCR10;CCR2
Neuroactive ligand-receptor interaction	2.80E-05	0.00258765	GLP1R;NPFFR1;CHRM1;SCT;PMCH;HTR2B;FPR3;LPAR3;TRH;ADRA1B;HTR6;HRH1;GALR2;C3AR1;CTSG;PRLH;DRD1;NTSR1;DRD4;PTGDR;P2RY13;EDN1;GZMA;F2R;TACR3;AGT;GRIN3B;GAL;SST;LEP;P2RX1;F2RL1;NMUR2;RLN3;AGTR2;AVP;F2RL2
Chemokine signaling pathway	5.36E-04	0.03097914	CXCL9;CCL11;CXCR5;CXCL1;PPBP;CXCL13;GNG12;GNG11;CXCL2;CX3CL1;CXCL5;GNG13;GNG10;GNG3;GRK7;CXCR3;XCL2;GNG8;CCR4;CRK;CCR10;CCR2
Taste transduction	5.59E-04	0.03097914	TAS2R60;HCN4;TAS2R31;TAS2R10;HTR3C;TAS2R13;TAS2R46;TAS2R19;GNG13;SCNN1G;CALHM1;TAS2R3;TAS1R3
IL-17 signaling pathway	0.00390301	0.18018912	CCL11;IL25;IFNG;CXCL1;DEFB4A;IL17D;CXCL2;TNF;MMP9;S100A9;CXCL5;IL17B
Glycosaminoglycan biosynthesis	0.00632545	0.25030706	CHST7;HS3ST3A1;B3GNT7;CHSY1;B3GNT2;CHSY3;HS6ST1;HS3ST2
Serotonergic synapse	0.01629697	0.55620104	CYP2C9;HTR6;GNG10;GNG3;PLA2G4D;KCNJ9;HTR2B;HTR3C;GNG8;GNG12;GNG11;GNG13

Amoebiasis	0.0188433	0.55620104	IL10;GNA14;IFNG;CXCL1;CTSG;SERPINB9;CD1D;LAMC1;TNF;CXCL2;TLR2
Inflammatory bowel disease	0.02073649	0.55620104	IL10;HLA-DRB5;MAF;IFNG;TBX21;TNF;IL2;TLR2
Asthma	0.02208741	0.55620104	IL10;HLA-DRB5;CCL11;RNASE3;TNF
Staphylococcus aureus infection	0.02836817	0.65483182	IL10;HLA-DRB5;DEFA6;KRT27;KRT14;KRT24;C3AR1;DSG1;FPR3;DEFB4A
Renin-angiotensin system	0.03076391	0.65550784	CPA3;CTSG;AGTR2;AGT
Caffeine metabolism	0.03630975	0.71841434	NAT2;CYP1A2
Breast cancer	0.04783646	0.7844821	TCF7L1;FZD4;WNT8B;WNT7A;FZD10;WNT9A;CCND1;FGF9;FGF18;E2F2;HES1;HES5;FGF10
Allograft rejection	0.04832079	0.7844821	IL10;HLA-DRB5;IFNG;TNF;IL2
JAK-STAT signaling pathway	0.04832916	0.7844821	IL10;EPO;LIF;OSM;IL2;IFNL1;CCND1;IFNG;LEP;IFNK;IL17D;SOCS4;IFNA21;SOCS5
Morphine addiction	0.05097718	0.7844821	GNG10;GNG3;KCNJ9;PDE3A;GNG8;DRD1;GNG12;GNG11;GNG13
Phototransduction	0.05788472	0.84389835	RCVRN;GUCY2D;GRK7;GUCA1B
Linoleic acid metabolism	0.06445317	0.89267646	CYP2C9;PLA2G12B;PLA2G4D;CYP1A2

Table S6. Enrichment analysis of features in muscle skeletal.

Term	P-value	Adjusted P-value	Genes
Cytokine-cytokine receptor interaction	5.86E-06	0.00128737	CXCL9;TNFRSF6B;CTF1;MSTN;EBI3;CXCR5;CXCL1;CXCR6;CXCL3;CXCL2;CX3CL1;CCL8;CXCR1;CCR10;CCR2;CCR1;GDF15;OSM;INHBB;PPBP;INHBC;BMP6;INHBE;TNFRSF10D;IL22RA1;BMP2;LEP;CD27;ACKR3;IL17D;CCL27;IL17B;TNFRSF21;PF4
Viral protein interaction with cytokine and cytokine receptor	9.36E-06	0.00128737	CCR1;CXCL9;CXCR5;CXCL1;PPBP;CXCL3;CXCL2;CX3CL1;TNFRSF10D;IL22RA1;CCL8;CXCR1;ACKR3;CCR10;CCL27;CCR2;PF4
Chemokine signaling pathway	2.70E-04	0.02474278	CCR1;CXCL9;CXCR5;CXCL1;PPBP;CXCR6;GNG12;CXCL3;CXCL2;CX3CL1;GNG10;GNG3;NRAS;CCL8;PIK3CA;CXCR1;GRK7;CRK;CCR10;CCL27;CCR2;PF4
Breast cancer	0.00115993	0.07974512	TCF7L1;NOTCH1;FZD5;FZD4;WNT8B;FZD10;WNT9A;FGF6;WNT6;NRAS;CCND1;FGF9;PIK3CA;FGF18;HES1;HES5;FGF10
Signaling pathways regulating pluripotency of stem cells	0.00225192	0.12385565	LEFTY1;FZD5;DLX5;FZD4;WNT8B;INHBB;FZD10;WNT9A;INHBC;INHBE;WNT6;NRAS;PIK3CA;ID3;HOXA1;MYF5
Basal cell carcinoma	0.01321736	0.48862331	WNT6;TCF7L1;BMP2;FZD5;FZD4;WNT8B;FZD10;WNT9A
Neuroactive ligand-receptor interaction	0.01349041	0.48862331	PTGER4;SCT;PTGER1;PTGER2;PMCH;OXT;ADRA1B;MCHR1;GALR3;UCN2;CHRNE;S1PR1;CHRNA10;INSL3;EDN1;P2RY1

			1;GZMA;F2R;SLURP1;GRIN3A;GAL;ADORA2B;SST;LEP;NMUR1;AVP;F2RL3
Glycosaminoglycan biosynthesis	0.01625191	0.48862331	CHST7;CHSY1;UST;B3GNT2;CHSY3;HS6ST1;HS3ST2
Gastric cancer	0.01803701	0.48862331	TCF7L1;FZD5;FZD4;WNT8B;FZD10;WNT9A;FGF6;WNT6;NRAS;CCND1;FGF9;PIK3CA;FGF18;FGF10
Acute myeloid leukemia	0.01869936	0.48862331	CEBPA;NRAS;TCF7L1;CCND1;PIK3CA;BCL2A1;CEBPE;EIF4EBP1
TGF-beta signaling pathway	0.01954493	0.48862331	GREM2;LEFTY1;BMP2;NOG;ID3;INHBB;INHBC;BMP6;RBX1;INHBE
Proteoglycans in cancer	0.02977895	0.68243431	FZD5;CAV3;FZD4;WNT8B;FZD10;WNT9A;HOXD10;MMP9;WNT6;NRAS;RRAS;CCND1;PIK3CA;KDR;TIMP3;TLR4;TLR2
Amoebiasis	0.03231884	0.68366768	GNA14;GNA15;PIK3CA;CXCL1;SERPINB9;CD1D;CXCL3;CXCL2;TLR4;TLR2
Pathways in cancer	0.0424483	0.80611229	PTGER4;CEBPA;FH;NOTCH1;PTGER1;PTGER2;WNT8B;FZD10;FGF6;WNT6;NRAS;GNG10;GNG3;FGF9;CCND1;PMAIP1;HES1;FADD;VHL;HES5;EDN1;TCF7L1;FZD5;FZD4;F2R;WNT9A;GNG12;MMP9;RBX1;BMP2;PIK3CA;MSH3;FGF18;CRK;F2RL3;FGF10
Rheumatoid arthritis	0.04396976	0.80611229	ATP6V1G1;HLA-DRB5;CXCL1;CXCL3;CXCL2;TLR4;HLA-DQA2;TLR2;ATP6V1F
Melanogenesis	0.06748752	0.9998734	WNT6;EDN1;NRAS;TCF7L1;FZD5;FZD4;WNT8B;FZD10;WNT9A
Circadian rhythm	0.06796389	0.9998734	BHLHE40;BHLHE41;NR1D1;RBX1
Melanoma	0.06927606	0.9998734	FGF6;NRAS;CCND1;FGF9;PIK3CA;FGF18;FGF10

Table S7. Enrichment analysis of features in nerve tibial.

Term	P-value	Adjusted P-value	Genes
Viral protein interaction with cytokine and cytokine receptor	2.06E-09	5.44E-07	IL10;CXCL9;CCL22;CXCR5;CXCR4;CXCL1;CXCL13;CXCL3;TNF;CXCL2;CX3CL1;CXCL5;TNFRSF10D;IL22RA1;CCL8;XCL2;ACKR4;ACKR3;CCR7;PF4V1;CCL27
Cytokine-cytokine receptor interaction	3.51E-08	4.63E-06	BMP10;CXCL9;CTF1;IL25;IL26;EPO;CXCR5;CXCR4;IL27;CXCL1;CXCR6;CXCL13;CXCL3;TNF;CXCL2;CX3CL1;CXCL5;CCL8;CCR7;PF4V1;IL10;CCL22;TNFSF15;GDF3;BMP6;TNFRSF10D;IL22RA1;BMP3;LEP;XCL2;ACKR4;ACKR3;IL17D;CCL27;IL17B;TNFRSF21
Neuroactive ligand-receptor interaction	2.76E-04	0.02424839	PTGER4;UCN;SCT;PTGER2;HTR2B;TRH;ADRA1B;HTR6;HRH1;UCN2;C3AR1;GNRHR;S1PR1;CTSG;DRD1;DRD4;PTGDR;NTSR2;AGT;SLURP1;TRHR;GRIN3A;GAL;ADORA2B;LEP;P2RX1;F2RL1;NMUR2;NMUR1;F2RL2
Staphylococcus aureus infection	9.57E-04	0.063157	IL10;HLA-DRB5;DEFA6;KRT27;KRT38;KRT26;DEFA4;KRT25;C3AR1;KRT12;KRT32;KRT20
Amoebiasis	0.00178666	0.0943355	IL10;GNA14;HSPB1;CXCL1;CTSG;SERPINB9;CD1D;LAMC1;CXCL3;TNF;CXCL2;TLR4
IL-17 signaling pathway	0.00284807	0.12531499	IL25;MMP1;CXCL1;FADD;CXCL3;IL17D;CXCL2;TNF;S100A9;CXCL5;IL17B

Chemokine signaling pathway	0.00509707	0.19223222	CXCL9;CCL22;CXCR5;CXCR4;CXCL1;CXCR6;CXCL13;GNG12;CXCL3;CXCL2;CX3CL1;CXCL5;CCL8;XCL2;CCR7;PF4V1;CCL27
Rheumatoid arthritis	0.00789423	0.26050969	ATP6V1G1;HLA-DRB5;MMP1;CXCL1;CXCL3;CXCL2;TNF;TLR4;CXCL5;ATP6V1F
Asthma	0.04610047	0.99956852	IL10;HLA-DRB5;RNASE3;TNF
Wnt signaling pathway	0.06437619	0.99956852	DKK4;TCF7L1;SFRP2;VANGL2;CCND1;SOX17;FZD5;FZD4;SFRP5;FZD10;SOST;WNT9A

Table S8. Enrichment analysis of features in skin.

Term	P-value	Adjusted P-value	Genes
Cytokine-cytokine receptor interaction	1.07E-10	3.04E-08	CXCL9;TNFRSF6B;CCL11;CTF1;IL25;MSTN;EBI3;CXCR4;IL27;CXCL1;CXCR6;CXCL13;CXCL3;TNF;CXCL2;CX3CL1;IFNL2;CCL8;CXCR3;CCR7;CCR4;CCR10;CCL22;GDF15;LIF;BMP8A;GDF3;PPBP;NGF;INHBC;IL22RA1;BMP3;BMP2;LEP;IL9;XCL2;ACKR4;CD27;XCL1;ACKR3;LTB;IL17D;INHA;CCL27;IL17B;TNFRSF21
Viral protein interaction with cytokine and cytokine receptor	1.58E-08	2.24E-06	CXCL9;CCL11;CCL22;CXCR4;CXCL1;PPBP;CXCL13;CXCL3;TNF;CXCL2;CX3CL1;IL22RA1;CCL8;CXCR3;XCL2;ACKR4;XCL1;ACKR3;CCR7;CCR4;CCR10;CCL27
Chemokine signaling pathway	1.68E-05	0.00158739	CXCL9;CCL11;CXCR4;CXCL1;CXCR6;CXCL13;CXCL3;CXCL2;CX3CL1;NRAS;CCL8;GRK7;CXCR3;GNG8;CCR7;CCR4;CCR10;CCL22;PPBP;GNG12;GNG11;GNG13;PIK3CA;XCL2;XCL1;CCL27
Neuroactive ligand-receptor interaction	2.46E-04	0.01745453	NPFFR1;PTGER1;NPY2R;CHRNA9;FPR3;OXT;FPR2;GRPR;ADRA1B;MCHR1;UCN2;CHRNE;GNRHR;S1PR1;CHRNA10;CTSG;S1PR3;DRD1;HCRT;NTSR1;DRD4;PTGDR;P2RY13;GZMA;F2R;SSTR1;AGT;SLURP1;GRIN3A;SST;LEP;NMUR1;AVP;F2RL2;F2RL3
Staphylococcus aureus infection	0.00188771	0.10722188	DEFA6;DEFA4;KRT34;DEFB1;FPR3;KRT32;FPR2;KRT28;KRT37;KRT14;DSG1;DEFB4A;HLA-DQA2
IL-17 signaling pathway	0.00494421	0.23402592	CCL11;IL25;MMP1;CXCL1;FADD;CXCL3;DEFB4A;IL17D;CXCL2;TNF;MMP9;IL17B
Amoebiasis	0.02291249	0.87922932	GNA14;PIK3CA;HSPB1;CXCL1;CTSG;CD1D;LAMC1;CXCL3;TNF;CXCL2;TLR2
Asthma	0.02476702	0.87922932	CCL11;IL9;RNASE3;TNF;HLA-DQA2
Type II diabetes mellitus	0.03750957	0.99828455	KCNJ11;PIK3CA;IRS1;MAFA;TNF;SOCS4
Caffeine metabolism	0.03837489	0.99828455	NAT2;CYP1A2
Rheumatoid arthritis	0.06633043	0.99828455	MMP1;CXCL1;LTB;CXCL3;CXCL2;TNF;HLA-DQA2;TLR2;ATP6V1F
TGF-beta signaling pathway	0.06999182	0.99828455	GREM2;LEFTY1;BMP2;BMP8A;ID3;FMOD;TNF;INHBC;RBX1

References

[1] J. Somekh, S. S. S. S. Shen-Orr, and I. S. I. S. Kohane, “Batch correction evaluation framework using a-priori gene-gene associations: Applied to the GTEx dataset,” *BMC Bioinformatics*, vol. 20, no. 1, p. 268, May 2019.
